# Supplementary material for: Deuteration-enhanced neutron contrasts to probe amorphous domain sizes in organic photovoltaic bulk heterojunction films
Source: Nat Commun. 2024 Mar 30;15:2784. doi: 10.1038/s41467-024-47052-7 (PMC10981694; doi:10.1038/s41467-024-47052-7)
Supplement: Supplementary file 1 — Supplementary Information [file 41467_2024_47052_MOESM1_ESM.pdf]

Supplementary Information

Deuteration-enhanced neutron contrasts to probe amorphous domain sizes in organic photovoltaic bulk heterojunction films

Guilong Cai[1,2,†], Yuhao Li[1,3,4,†*], Yuang Fu[1,†], Hua Yang[3,4], Le Mei[5], Zhaoyang Nie[6], Tengfei Li[7], Heng Liu[1], Yubin Ke[3,4], Xun-Li Wang[8,9], Jean-Luc Brédas[10], Man-Chung Tang[6], Xiankai Chen[5], Xiaowei Zhan[7,*] and Xinhui Lu[1,*]

[1] Department of Physics, The Chinese University of Hong Kong, Hong Kong, China. E-mail: xinhui.lu@cuhk.edu.hk

[2] Beijing Key Laboratory of Ionic Liquids Clean Process, Institute of Process Engineering, Chinese Academy of Sciences, Beijing, China.

[3] Spallation Neutron Source Science Center, Dongguan 523803, China. E-mail: liyuhao@ihep.ac.cn

[4] Institute of High Energy Physics, Chinese Academy of Sciences, Beijing 10049, China.

[5] Department of Materials Science and Engineering, City University of Hong Kong, Hong Kong, China.

[6] Institute of Materials Research, Tsinghua Shenzhen International Graduate School, Tsinghua University, Shenzhen 518055, China.

[7] School of Materials Science and Engineering, Peking University, Beijing, China. E-mail: xwzhan@pku.edu.cn

[8] Department of Physics and Center for Neutron Scattering, City University of Hong Kong, Hong Kong, China.

[9] Hong Kong Institute for Advanced Study, City University of Hong Kong, Hong Kong, China.

[10] Department of Chemistry and Biochemistry, The University of Arizona, Tucson, Arizona 85721-0041, USA.

† These authors contributed equally: Guilong Cai, Yuhao Li, Yuang Fu

* Corresponding authors

Supplementary Notes

Supplementary Note 1. Synthesis and characterizations of *d*-Y6 and *d*-Y7.

Compound S2. To the round bottom flask (25 mL) charged with dodecanoic-D23 acid (2.0 g, 9.0 mmol) under nitrogen atmosphere, thionyl chloride ($SOCl_2$) (1.3 mL, 18.0 mmol) was added. The reaction mixture was placed into preheated oil bath at 60 ℃. After 5 minutes the nitrogen balloon was changed, due to hydrogen chloride gas released in the reaction, this process was continued until there is no more hydrogen chloride gas evolved from the reaction. Excess thionyl chloride was removed under reduced pressure and dried over high vacuum for overnight. The residue Compound S1 is used in next step without further purification. To a stirring mixture solution of 3-bromothiophene (1.3 g, 8.0 mmol) and Compound 1 (2.0 g, 8.0 mmol) in CH_2Cl_2 (30 mL), $AlCl_3$ (1.0 g, 8.0 mmol) was added in portions over 15 min. The resulting solution was stirred for 3 h at room temperature. Cold water (20 mL) was then slowly added to quench the reaction. The crude product was extracted from the mixture solution with CH_2Cl_2 (3 × 20 mL). The combined organic layers were washed with brine and dried over anhydrous $MgSO_4$. Removal of the organic solvent gives the colorless liquid product of Compound S2 (3.8g, 84%). ^{1}H NMR (400 MHz, Chloroform-*d*) δ 7.5 (d, J = 5.2 Hz, 1H), 7.1 (d, J = 5.2 Hz, 1H).

Compound S4. To a stirring mixture solution of Compound S2 (2.0 g, 5.0 mmol) and K_2CO_3 (1.6 g, 11.0 mmol) in DMF (30 mL), ethyl thioglycolate (0.58 mL, 5.3 mmol)

was added. The resulting mixture solution was stirred for 24 h at ambient temperature. A solution of NaOH (0.05 g, 1.3 mmol) in ethanol (3 mL) was added, and the reaction was continued for another 24 h. The reaction mixture was poured into water (50 mL) and extracted with ethyl acetate (3 × 20 mL). The organic layer was washed with water (3 × 20 mL) and dried over anhydrous $MgSO_4$. The crude product of Compound S3 was obtained by removing organic solvents and used directly for the next step without further purification. To a mixture solution of Compound S3 (1.5 g, 1.5 mmol) in ethanol (30 mL), NaOH (0.16 g, 4.0 mmol) was added. The resulting solution was heated to reflux for ca. 3 h. After starting materials was completely consumed by TLC indication, the solvent was removed in vacuo. 30 mL of water was added to the residue. The pH was adjusted to 1 with 10 M HCl to precipitate the titled product. Recrystallization from hexane gave the product Compound S4 (1.1g, 80%). ^{1}H NMR (400 MHz, Chloroform-*d*) δ 7.5 (d, J = 5.2 Hz, 1H), 7.1 (d, J = 5.2 Hz, 1H), 2.8 (s, 2H).

Compound S5. A mixture of Compound S4 (1.0 g, 2.8 mmol), copper (0.09 g, 1.46 mmol), and quinoline (5 mL) was heated to reflux for 3 h. The resultant solution was concentrated by vacuum distillation. Ethyl acetate (20 mL) was added, and the collected organic layer was washed with 1 M HCl (3 × 15 mL) and dried over anhydrous $MgSO_4$. The solvent was removed in vacuo, and the residue was passed through a silica gel column eluting with hexane to yield Compound S5 (550 mg, 63%). ^{1}H NMR (400 MHz, Chloroform-*d*) δ 7.4 (dd, J = 5.2, 1.6 Hz, 1H), 7.2 (d, J = 5.2 Hz, 1H), 7.0 (d, J = 1.3 Hz, 1H), 2.7 (s, 2H).

Compound 1. Compound S5 (500 mg, 1.6 mmol) was dissolved in anhydrous tetrahydrofuran (THF, 20 mL) in an argon atmosphere, and stirred at −78 °C for 30 min. *n*-butyllithium (1.6 mmol, 2.5 mol/L, 0.64 mL) was added into the solution by dropwise at −78 °C and continued to stir for 2 h. Tributyltin chloride (220 mg, 0.67 mmol) was dripped in the mixture. After stirring for 30 min, the mixture was moved to room temperature and reacted overnight. Then, the mixture was extracted with dichloromethane, concentrated in vacuo, and dried to produce crude product. Then, redissolve the crude product in hexanes and filter, and condense the filtrate to obtain the Compound 1 (820 mg, 85%). ^{1}H NMR (400 MHz, Chloroform-*d*) δ 7.2 (s, 1H), 6.9 (s, 1H), 2.7 (s, 2H), 1.7 – 1.5 (m, 6H), 1.4 – 1.3 (m, 6H), 1.2 – 1.0 (m, 6H), 0.9 (t, J = 7.3 Hz, 9H).

Compound S7. To a solution of Compound S6 (1.5 g, 10 mmol) in acetic anhydride (10 mL) and triethylamine (5.0 mL) was added ethyl acetoacetate (1.4 mL, 11.0 mmol) under argon. The mixture was stirred at room temperature for 12 h, and then the mixture was poured into the mixture of concentrated HCl (10 g) and ice-water (10 g). After that, the mixture was stirred for half an hour at room temperature, additional HCl (18.0 mL, 5M) was added into the mixture, and then the mixture was refluxed for 2 h. After the crude product was cooled to room temperature, add water (100 mL) and then the mixture was extracted with $CHCl_3$ (3 × 50 mL). The organic phase was dried over anhydrous $MgSO_4$. After removing the solvent, the residue was purified using column chromatography on silica gel employing $CHCl_3$ as an eluent, yielding a pale yellow

solid (755 mg, 51%). ^{1}H NMR (400 MHz, Chloroform-*d*) δ 3.25 (s, 2H).

***d*-IC.** To a solution of Compound 6 (150 mg, 1.0 mmol) and malononitrile (130 mg, 2.0 mmol) in ethanol (20 mL) was added sodium acetate (107 mg, 1.3 mmol). The mixture was stirred for 2 h at room temperature. A volume of 30 mL of H_2O was added, and the mixture was stirred at room temperature for half an hour, and then a drop of concentrated HCl was added to acidify the mixture with PH = 2. After that, the brown solid was filtered off and washed with water (4 × 100 mL). The residue was purified using column chromatography on silica gel employing $CH_3OH/CHCl_3$ (1:1, v/v) as an eluent, yielding a brown solid (118 mg, 46.6%). ^{1}H NMR (400 MHz, Acetone-*d*6) δ 3.31 (s, 2H).

Compound S9. Synthesis of Compound S9 was carried out in a similar manner to that of Compound S7. ^{1}H NMR (400 MHz, Chloroform-*d*) δ 3.26 (s, 2H).

***d*-2FIC.** Synthesis of Compound *d*-2FIC was carried out in a similar manner to that of *d*-IC. ^{1}H NMR (400 MHz, Chloroform-*d*) δ 3.75 (s, 2H).

Compound 3. Compound 1 (2.7 g, 4.4 mmol), compound 2 (0.8 g, 2.0 mmol) and $Pd(PPh_3)_2Cl_2$ (62 mg,0.09 mmol) were dissolved in 30 mL of dry toluene and stirred at 80 °C overnight. The reaction mixture was allowed to cool to room temperature. 100 mL of water was added, and the mixture was extracted with dichloromethane (2 × 100 mL). The precipitate was filtered and washed with methanol and *n*-hexane. The product of Compound 3 was obtained as a red solid.

Compound 4. Compound 3 (850 mg, 1.0 mmol) and triphenylphosphine (3.2 g, 10.0

mmol) were dissolved in the *o*-dichlorobenzene (15 mL) under nitrogen. After being heated at 180 °C overnight, the aqueous phase was extracted with dichloromethane and the organic layer was dried over Na_2SO_4 and filtered. After removing the solvent, the red residue was added into a three-necked round bottom flask. 1-Bromo-2-ethylhexane-D17 (3.0 g, 1.5 mmol), KI (1.3 g, 7.8 mmol), K_2CO_3 (3.2 g, 23.2 mmol) and DMF (30 mL) were added and the mixture was deoxygenated with argon for 15 min. The mixture was refluxed at 80 °C for overnight. After removing the solvent from the filtrate, the residue was extracted with ethyl acetate and H_2O. The organic layers were combined and dried over $MgSO_4$, filtered and purified with column chromatography on silica gel using *n*-hexane/dichloromethane (10/1, v/v) as the eluent to give a red solid (532 mg, 51%).^{1}H NMR (400 MHz, Chloroform-*d*) δ 7.0 (s, 2H), 2.8 (s, 4H). MS (MALDI-TOF) calcd for $C_{56}H_6D_{76}N_4S_5$: 1046.9913; found: 1046.5179 (M^+).

Compound 5. A Vilsmeier reagent was prepared firstly. 8 mL anhydrous DMF was added to a dry 100 mL two-necked round bottom flask, and the solution was cooled to 0 °C and stirred when 0.8 mL phosphorous oxychloride ($POCl_3$) was added by syringe under argon protection. The mixture kept at 0 °C for 2 h, and then Compound 4 (420 mg, 0.4 mmol) in dry 1,2-dichloroethane (25 mL) was added. Then, the mixture solution was allowed to reflux overnight. After cooling to room temperature, 100 mL water was added to quench the reaction. The mixture was extracted with dichloromethane, and the organic layer was collected, washed with water and dried with anhydrous $MgSO_4$. After removal of the solvent under reduced pressure, the residue

was purified by column chromatography on silica gel using a mixture solvent as eluent (petroleum ether/dichloromethane, v/v = 1/1) to give a yellow solid (389 mg, 88%). ^{1}H NMR (400 MHz, Chloroform-*d*) δ 10.1 (s, 2H), 3.2 (s, 4H). MS (MALDI-TOF) calcd for $C_{58}H_6D_{76}N_4O_2S_5$: 1102.9811; found: 1102.3838 (M^+).

***d*-Y6.** To a three-necked round bottom flask were added Compound 5 (220 mg, 0.2 mmol), *d*-2FIC (140 mg, 0.6 mmol), pyridine (0.15 mL) and chloroform (25 mL). The mixture was deoxygenated with nitrogen for 20 min and then stirred at reflux for 12 h. After cooling to room temperature, the mixture was poured into methanol (200 mL) and filtered. The residue was purified by column chromatography on silica gel using a mixture solvent as eluent (petroleum ether/dichloromethane, v/v = 1/1) to give a blue solid (255 mg, 83%). ^{1}H NMR (400 MHz, Chloroform-*d*) δ 9.2 (s, 2H), 3.2 (s, 4H). ^{13}C NMR (100 MHz, Chloroform-*d*) δ 186.3, 159.0, 154.1, 147.7, 145.3, 137.9, 136.1, 135.4, 134.6, 134.0, 133.3, 130.7, 120.1, 115.1, 114.7, 113.7, 68.9, 29.8. The presence of C-F coupling is evidenced by the doublet at δ 155.64 (d, *J*=16.2 Hz), 153 (dd, *J*=14.8, 7.3 Hz). HRMS (MALDI) calculated for $C_{82}H_6D_{80}F_4N_8O_2S_5$: 1531.0429; found: 1531.0424 (M^+). Elemental analysis calculated for $C_{82}H_7D_{80}F_4N_8O_2S_5$: C, 64.23; N, 7.31. Found: C, 64.12; N, 7.13.

***d*-Y7** The material was synthesized following the same route as *d*-Y6. ^{1}H NMR (400 MHz, Chloroform-*d*) δ 9.17 (s, 1H), 8.79 (s, 1H), 7.97 (s, 1H), 3.21 (s, 2H) ppm; ^{13}C NMR (151 MHz, Chloroform-*d*) δ 186.42, 158.89, 154.37, 147.68, 145.46, 139.71, 139.31, 138.90, 137.93, 136.38, 136.21, 135.97, 134.04, 133.70, 131.01, 127.06,

125.16, 120.08, 115.19, 114.71, 113.81, 68.90, 29.80, 29.47 ppm.

Supplementary Note 2. Synthesis and characterizations of *d*-IDIC.

Compound S11. To a suspension of Compound S10 (500 mg, 1.9 mmol) in anhydrous DMSO (15 ml) was added potassium tert-butoxide (1.2 g, 11.3 mmol) in portion. The reaction mixture was heated at 80 °C for 1 h, followed by the addition of 1-Bromohexane-D13 (2.0 g, 11.3 mmol) dropwise. After complete addition, the resultant mixture was heated at 90 °C for 5 h, then poured into ice-water. The resulting mixture was extracted with chloroform (3 × 50 mL) and the combined organic extracts were washed with brine. The organic mixture was dried over anhydrous magnesium sulfate. After filtration, the solvent was removed by rotary evaporation to give a brown liquid. The crude product was purified by column chromatography using hexane/dichloromethane (v/v = 4:1) as the eluent to afford pale yellow solid (1.1 g, 87%). ^{1}H NMR (500 MHz, Chloroform-*d*) δ 7.20 (s, 2H), 7.18 (d, J = 4.6 Hz, 2H), 6.89 (d, J = 4.8 Hz, 2H). MS (MALDI-TOF) calcd for $C_{40}H_6D_{52}S_2$: 654.7244; found: 654.7194 (M^+).

Compound S12. A Vilsmeier reagent was prepared firstly. 8 mL anhydrous DMF was added to a dry 100 mL two-necked round bottom flask, and the solution was cooled to 0 °C and stirred when 0.8 mL phosphorous oxychloride ($POCl_3$) was added by syringe under argon protection. The mixture kept at 0 °C for 2 h, and then Compound S11 (280 mg, 0.4 mmol) in dry 1,2-dichloroethane (25 mL) was added. Then, the mixture

solution was allowed to reflux overnight. After cooling to room temperature, 100 mL water was added to quench the reaction. The mixture was extracted with dichloromethane, and the organic layer was collected, washed with water and dried with anhydrous $MgSO_4$. After removal of the solvent under reduced pressure, the residue was purified by column chromatography on silica gel using a mixture solvent as eluent (petroleum ether/dichloromethane, v/v = 1/1) to give a yellow solid (270 mg, 89%). ^{1}H NMR (500 MHz, Chloroform-*d*) δ 9.9 (s, 2H), 7.6 (d, J = 1.4 Hz, 2H), 7.5 (s, 2H). MS (MALDI-TOF) calcd for $C_{42}H_6D_{52}O_2S_2$: 710.7142; found: 710.6473 (M^+).

***d*-IDIC.** To a three-necked round bottom flask were added Compound S12 (142 mg, 0.2 mmol), *d*-IC (120 mg, 0.6 mmol), pyridine (0.15 mL) and chloroform (25 mL). The mixture was deoxygenated with nitrogen for 20 min and then stirred at reflux for 12 h. After cooling to room temperature, the mixture was poured into methanol (200 mL) and filtered. The residue was purified by column chromatography on silica gel using a mixture solvent as eluent (petroleum ether/dichloromethane, v/v = 1/1) to give a blue solid (173 mg, 81%). ^{1}H NMR (400 MHz, Chloroform-*d*) δ 9.0 (s, 2H), 7.7 (s, 2H), 7.6 (s, 2H). ^{13}C NMR (100 MHz, Chloroform-*d*) δ 188.5, 160.9, 160.4, 157.7, 156.8, 141.3, 140.1, 138.7, 138.1, 137.9, 137.1, 122.2, 116.2, 115.0, 114.9, 69.1, 54.2. HRMS (MALDI) calculated for $C_{66}H_6D_{60}N_4O_2S_2$: 1070.8388; found: 1070.8384 (M^+). Elemental analysis calculated for $C_{66}H_6D_{60}N_4O_2S_2$: C, 73.96; N, 5.23. Found: C, 73.56; N, 5.07.

Supplementary Figures

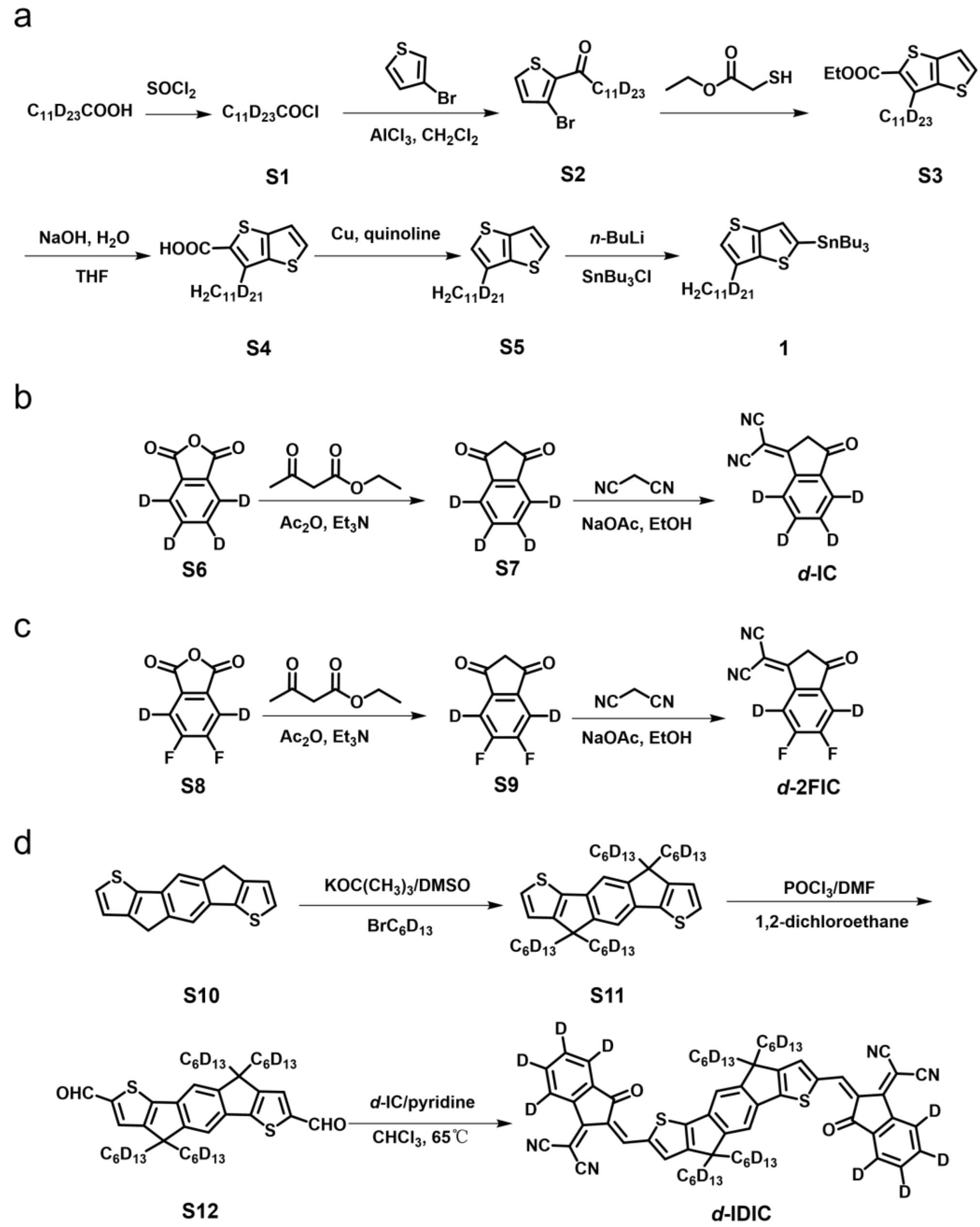

Supplementary Fig. 1 Synthetic routes. Synthetic routes of **a** Compound 1, **b** *d*-IC, **c** *d*-2FIC, and **d** *d*-IDIC.

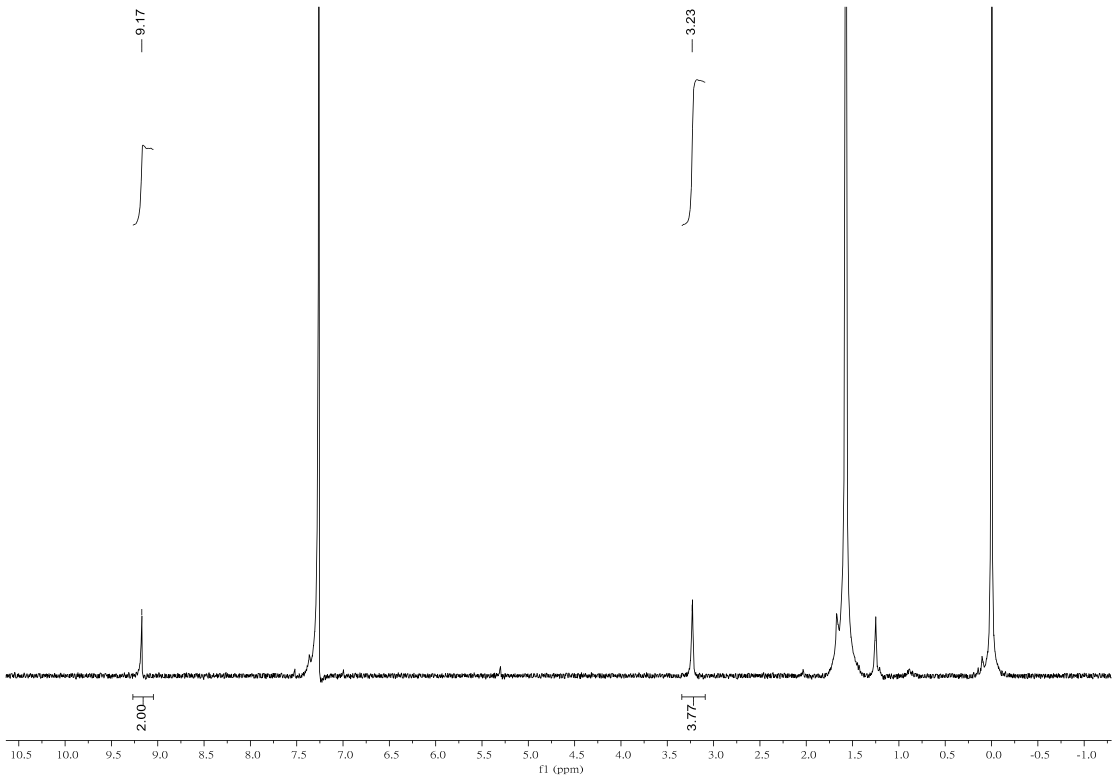

Supplementary Fig. 2 ^{1}H NMR spectrum of *d*-Y6.

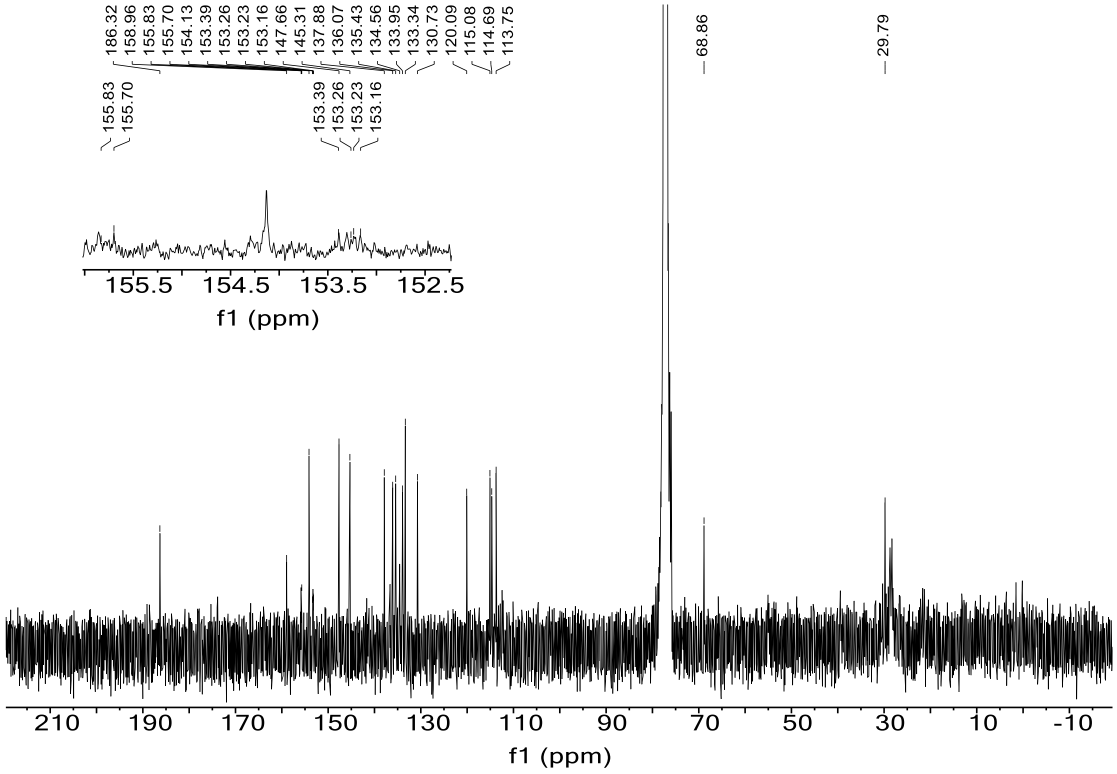

Supplementary Fig. 3 ^{13}C NMR spectrum of *d*-Y6. The inset is the enlarged spectrum between 152 and 156 ppm to highlight the presence of C-F coupling.

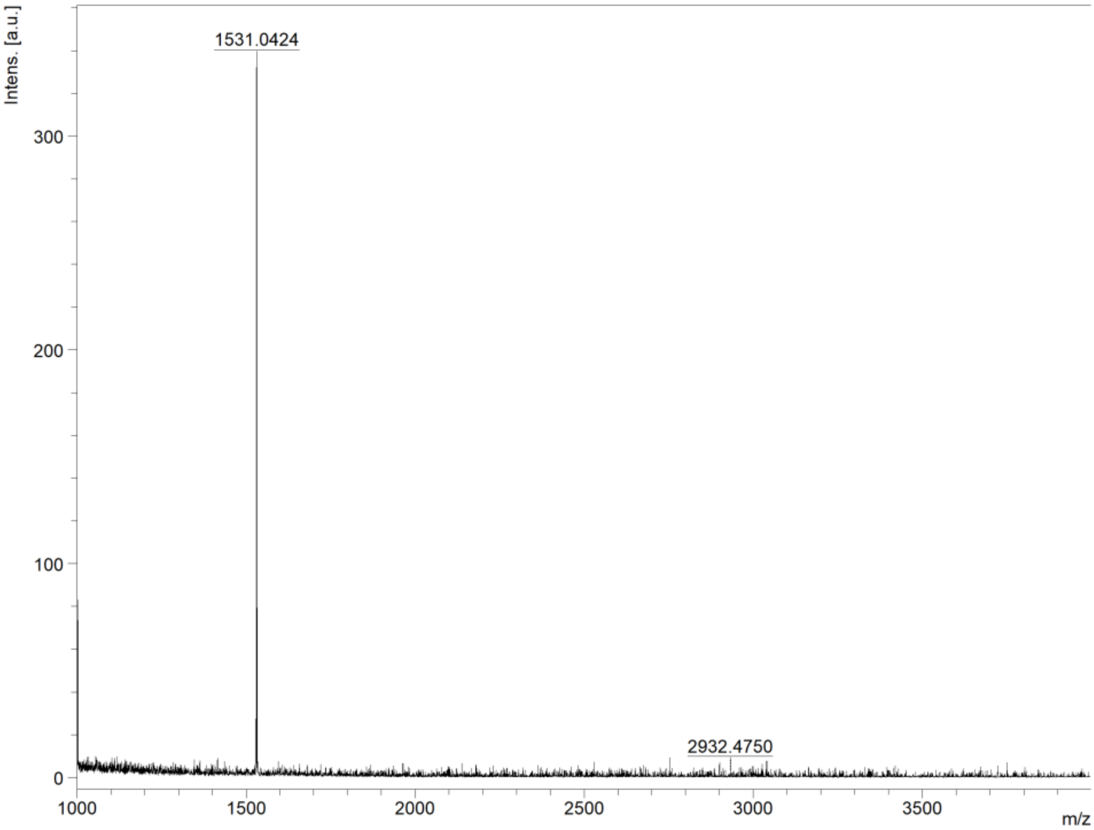

Supplementary Fig. 4 HRMS (MALDI) spectrum of *d*-Y6.

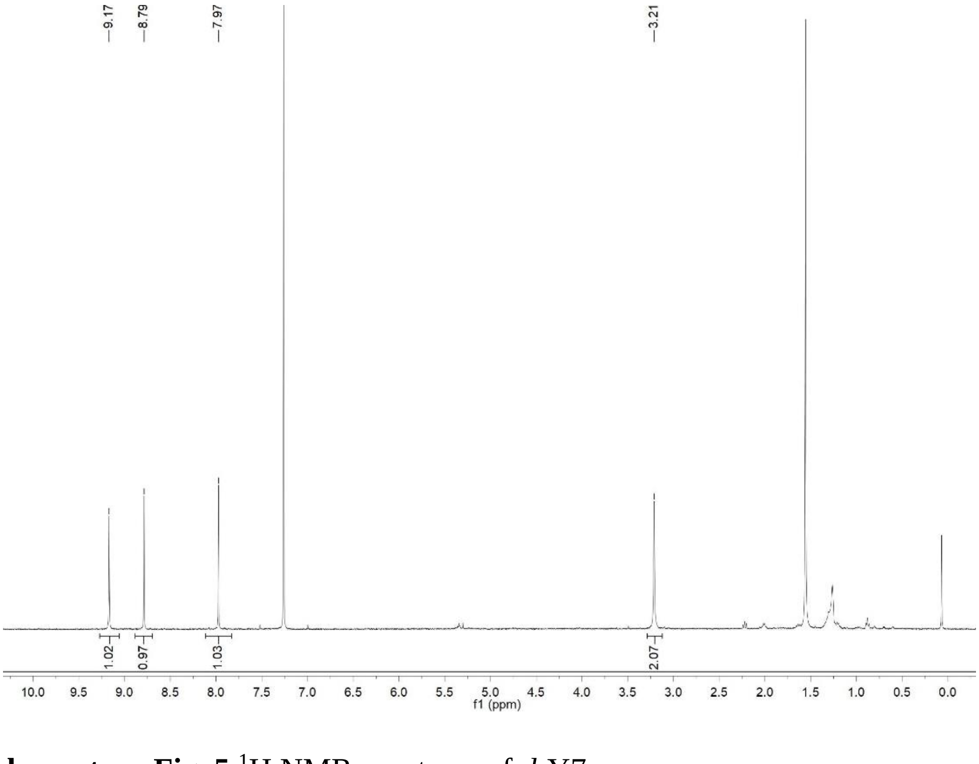

Supplementary Fig. 5 ^{1}H NMR spectrum of *d*-Y7.

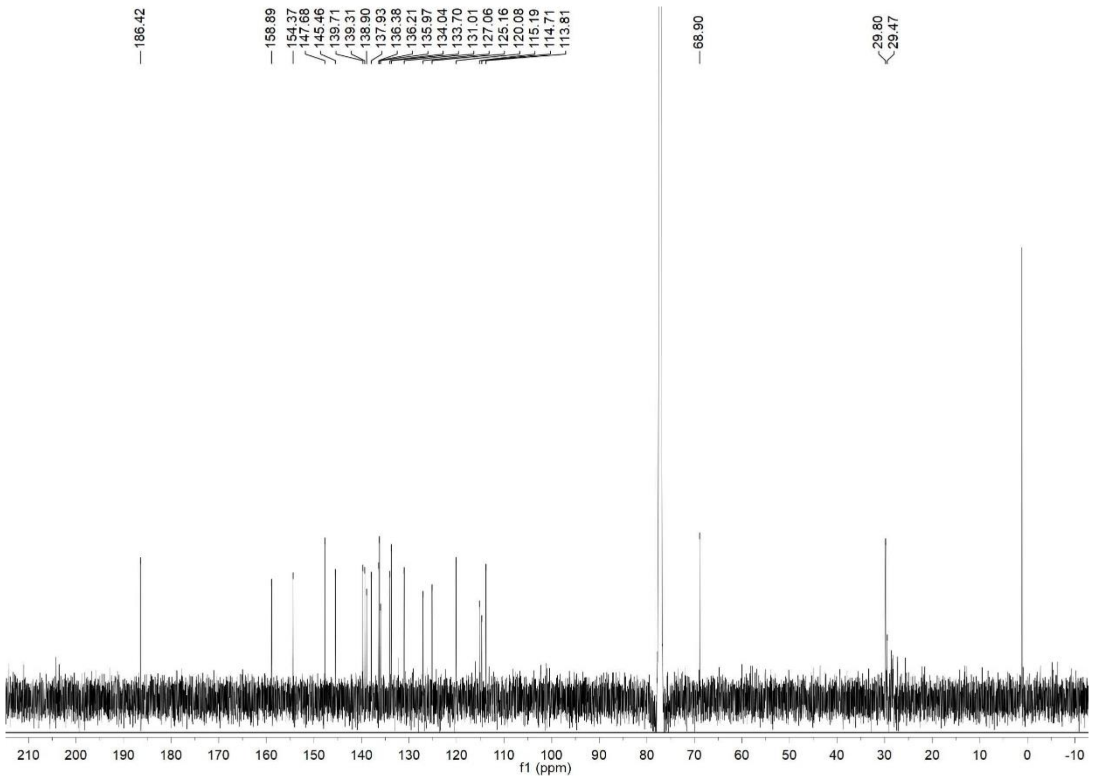

Supplementary Fig. 6 ^{13}C NMR spectrum of *d*-Y7.

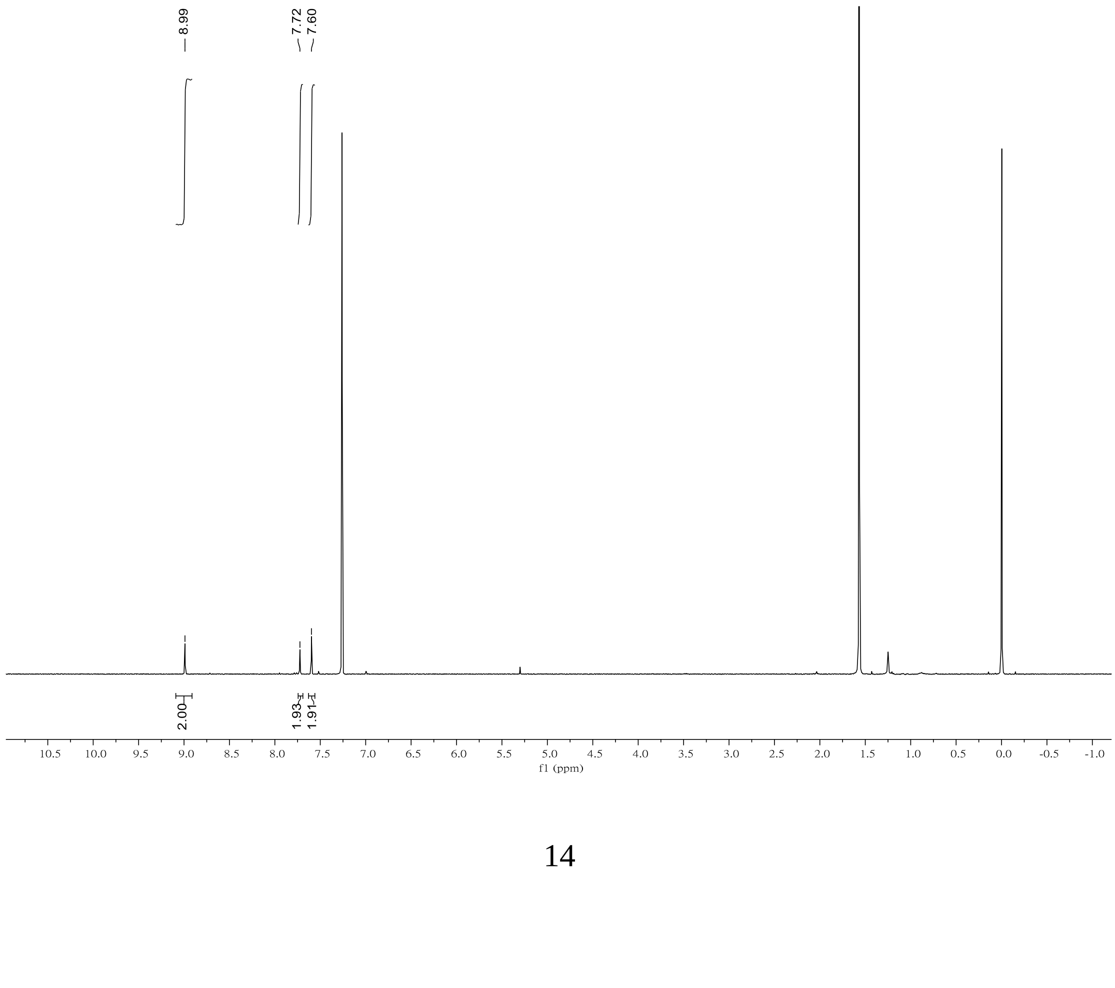

Supplementary Fig. 7 ^{1}H NMR spectrum of *d*-IDIC.

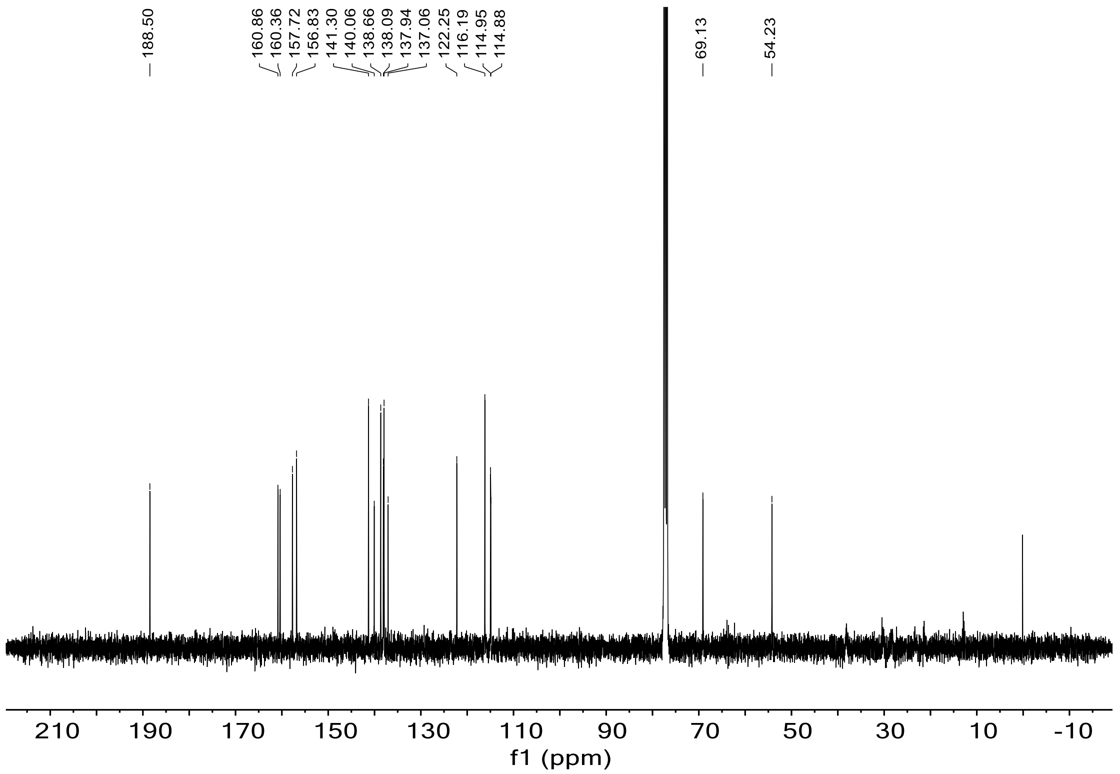

Supplementary Fig. 8 ^{13}C NMR spectrum of *d*-IDIC.

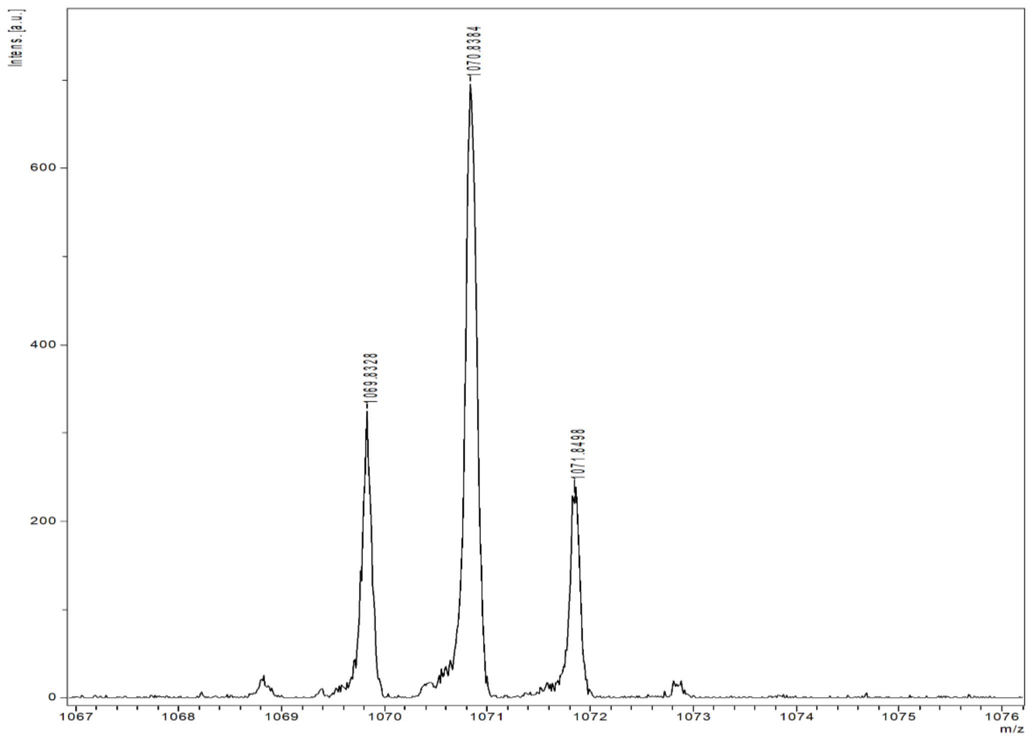

Supplementary Fig. 9 HRMS (MALDI) spectrum of *d*-IDIC.

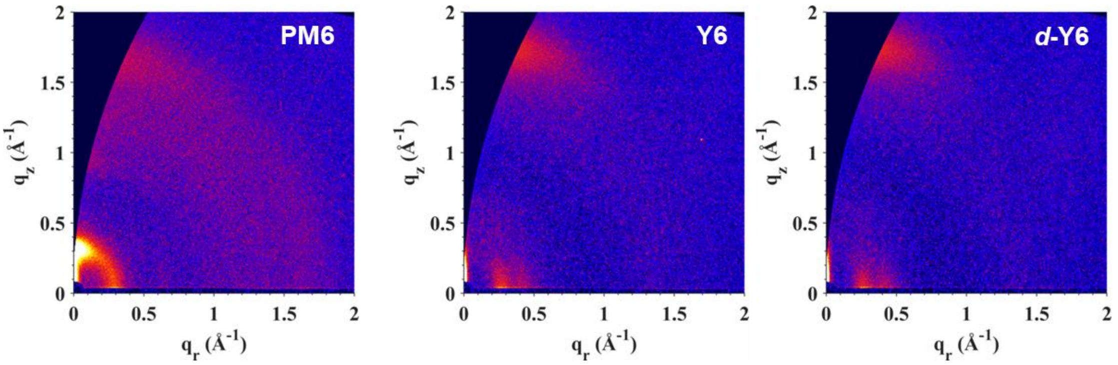

Supplementary Fig. 10 2D GIWAXS patterns of pure PM6, Y6, and *d*-Y6 films.

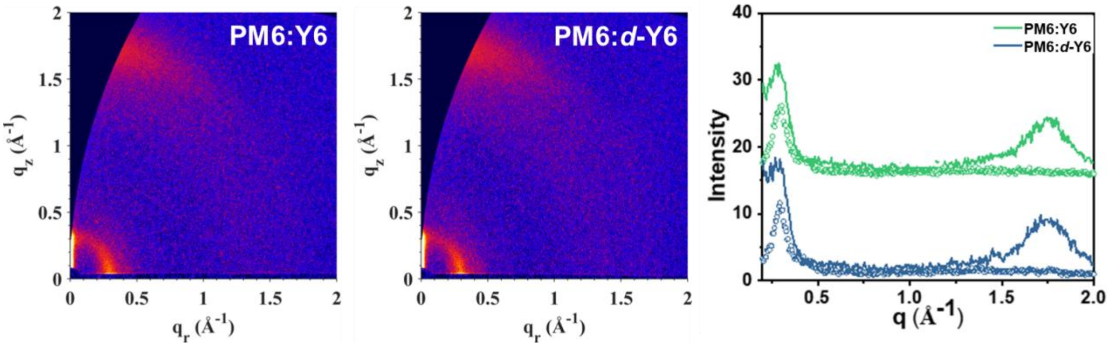

Supplementary Fig. 11 2D GIWAXS patterns and intensity profiles along the in-plane (dashed line) and out-of-plane (solid line) directions of PM6:Y6 and PM6:*d*-Y6.

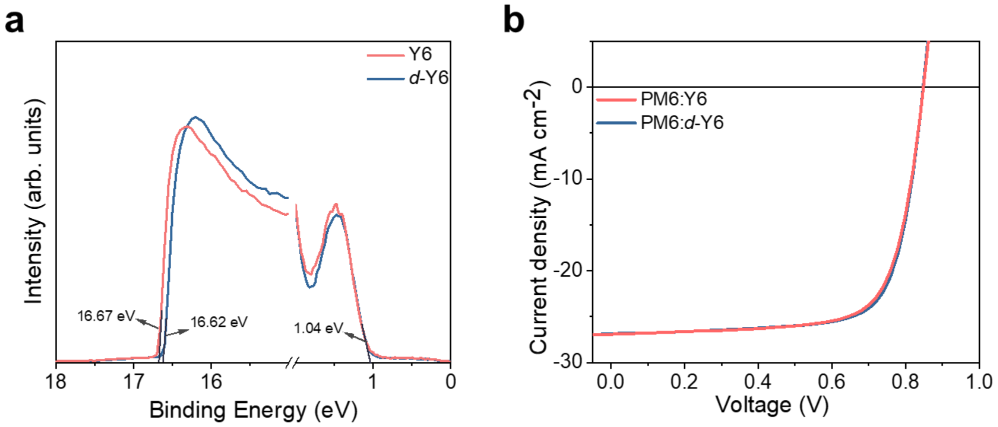

Supplementary Fig. 12 a UPS spectra of PM6:Y6 and PM6:*d*-Y6 blend films and **b** *J*–*V* curves of the corresponding devices.

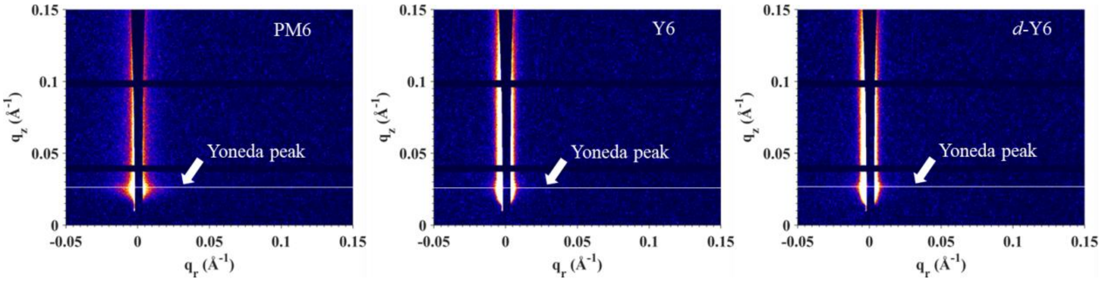

Supplementary Fig. 13 2D GISAXS patterns of pure PM6, Y6, and d-Y6 films measured with the vertical beam stop. The Yoneda peak was marked by the white line for each film.

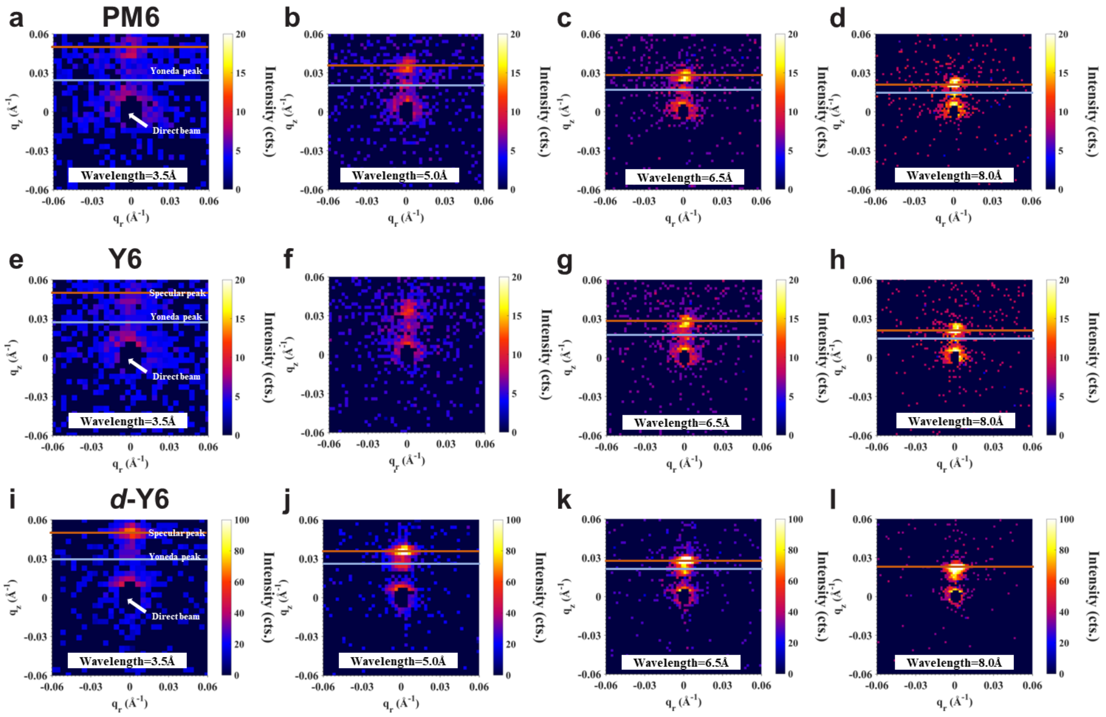

Supplementary Fig. 14 2D TOF-GISANS data of **a-d** pure PM6, **e-h** Y6 and **i-l** *d*-Y6 films with different neutron wavelengths, taken at the same incident angle of 0.804°.

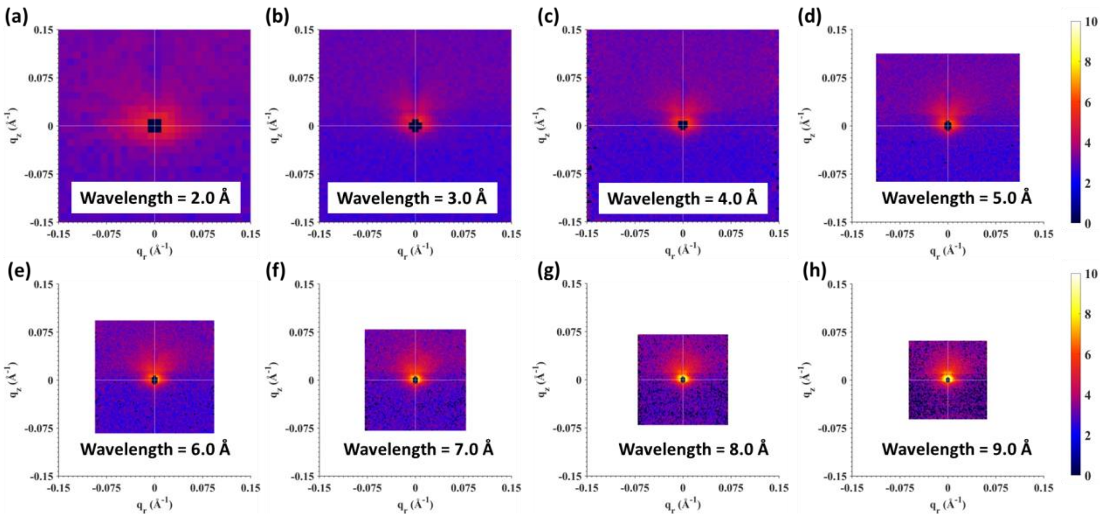

Supplementary Fig. 15 2D TOF-GISANS patterns of the PM6:Y6 blend film measured at wavelengths of **a-h** 2-9 Å. All measurements were taken at the same incident angle of 0.606°.

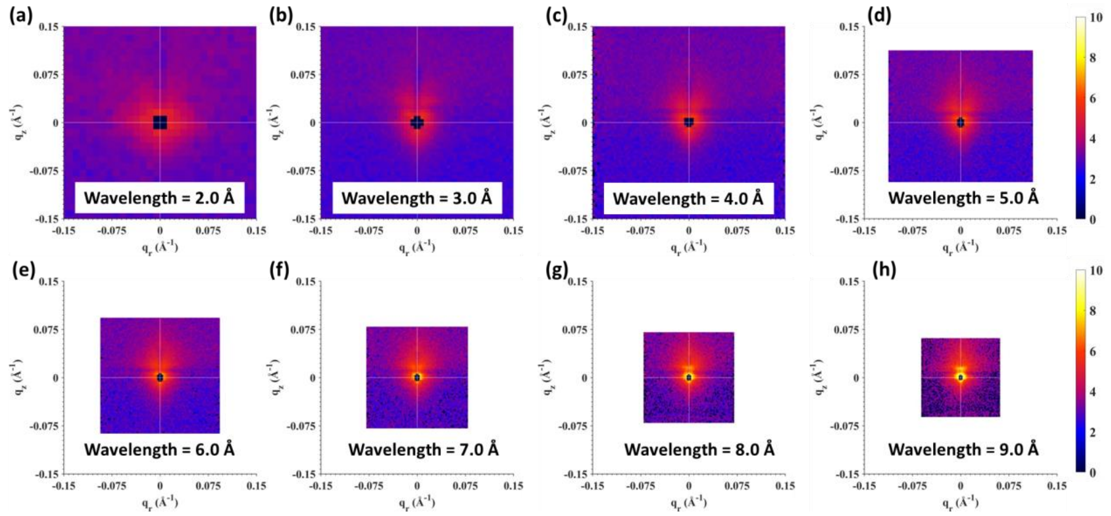

Supplementary Fig. 16 2D TOF-GISANS patterns of the PM6:*d*-Y6 blend film measured at wavelengths of **a-h** 2-9 Å. All measurements were taken at the same incident angle of 0.606°.

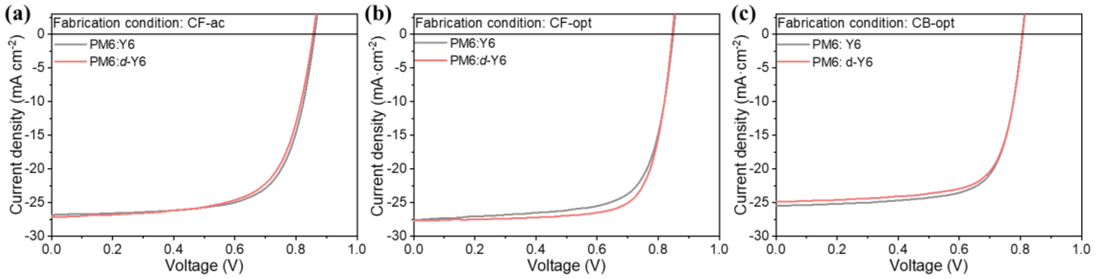

Supplementary Fig. 17 Typical J-V curves of the devices based on PM6:Y6 and PM6:*d*-Y6 under three different conditions (**a** CF-ac, **b** CF-opt, **c** CB-opt) mentioned in the main text.

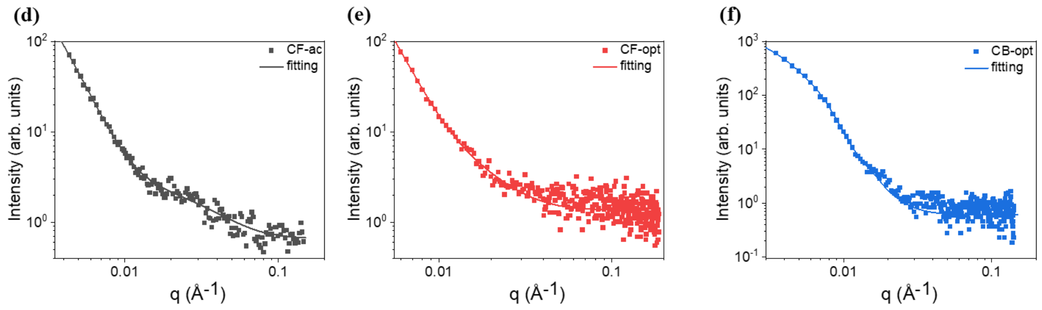

Supplementary Fig. 18 Horizontal linecuts along the in-plane direction (dotted lines) extracted from the 2D GISAXS patterns of **a** PM6: *d*-Y6 as cast, **b** CF-optimized, and **c** CB-optimized blend films with their best fittings (solid lines).

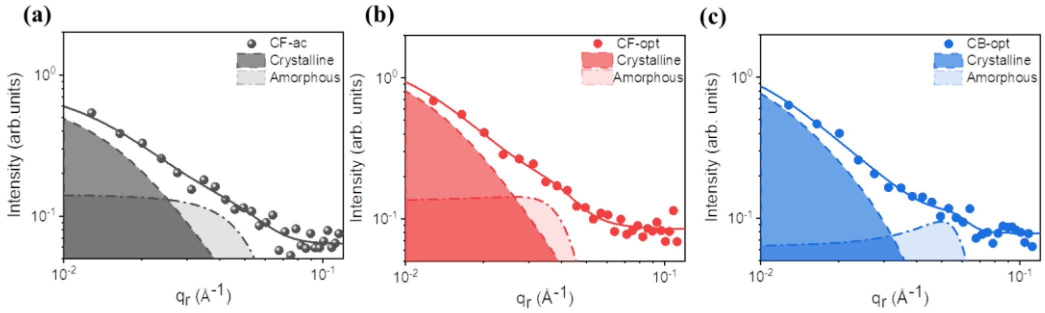

Supplementary Fig. 19 Horizontal linecuts along the in-plane direction (dotted lines) extracted from the 2D GISANS patterns of **a** PM6: *d*-Y6 as cast, **b** CF-optimized, and **c** CB-optimized blend films with their best fittings (solid lines).

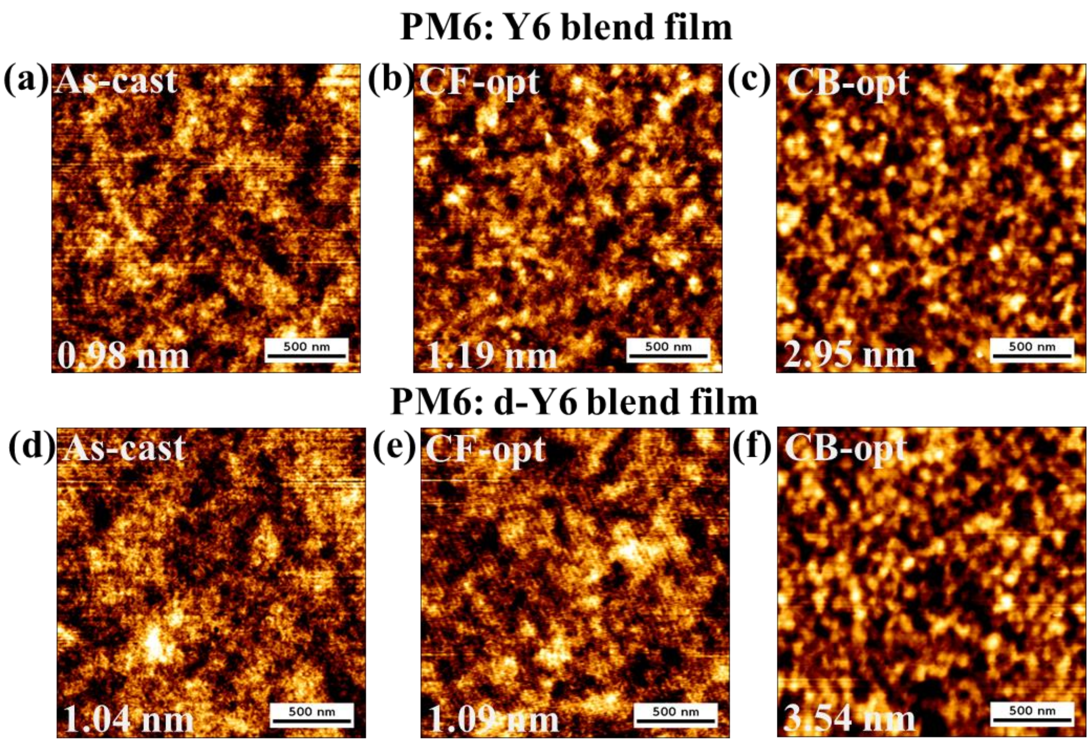

Supplementary Fig. 20 AFM topography images of PM6:Y6 (**a**-**c**) and PM6: *d*-Y6 (**d**-**f**) blend films processed under different conditions mentioned in the main text. The root-mean-square (RMS) roughness of heights is labelled at the inset for each image.

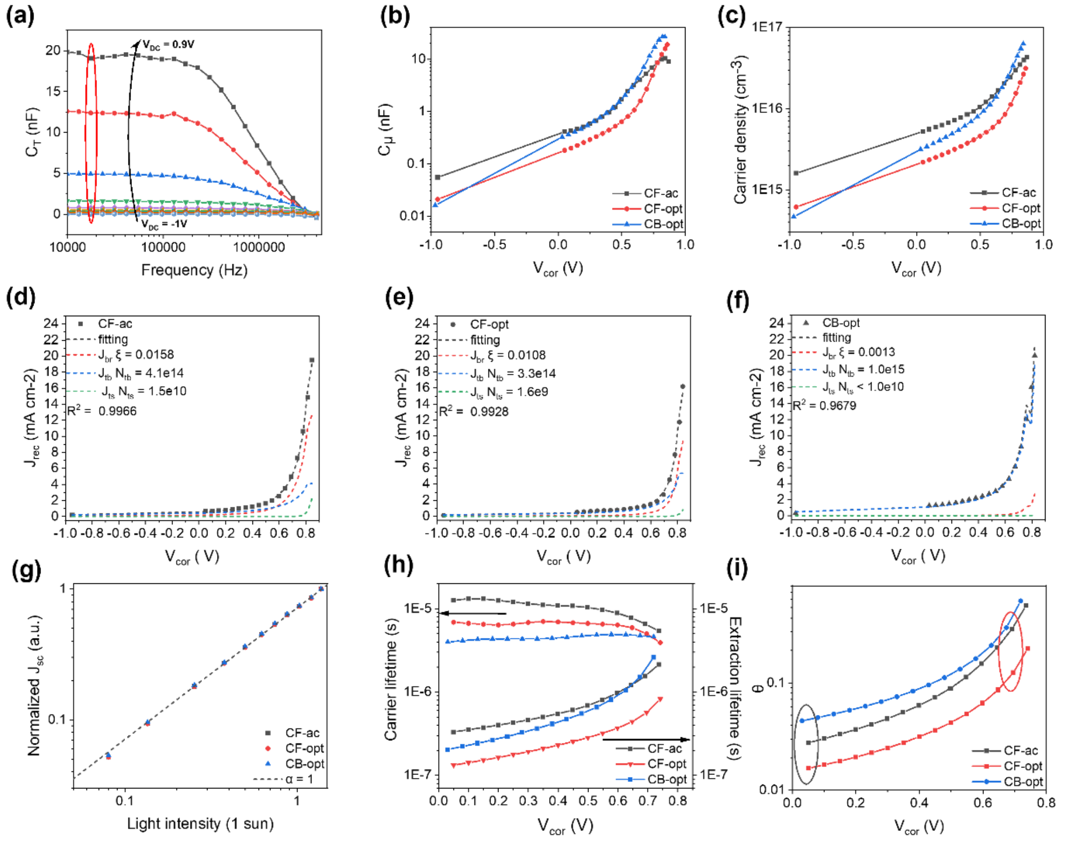

Supplementary Fig. 21 **a** The total capacitance associated with the active layer (C_T) of the CF-optimized device as a function of the A.C. frequency applied, corrected for geometric capacitance. V_{DC} is the background bias applied to the device under 1 sun illumination, which increases from –1 V to 0.9 V. Chemical capacitance (C_μ) is taken at a low frequency of 17 kHz where the C_T saturates, as highlighted in red. **b** C_μ, **c** charge carrier density stored within the active layer as a function of applied bias after correcting for series resistance (V_{cor}). The fits of the recombination current of **d** as-cast, **e** CF-optimized, and **f** CB-optimized PM6:Y6 device via superposition of bimolecular, bulk, and surface-trap assisted recombination as discussed in the main text. **g** the intensity-dependent J_{sc} measurements for the three devices. **h** the charge carrier lifetime $\tau = \frac{qnL}{J_{rec}}$ and charge extraction time $\tau_{ext} = \frac{qnL}{J}$ as a function of V_{cor}, where q is the elementary charge, n is the charge carrier density, L is the active layer thickness and J_{rec} and J are recombination current density and current density under light,

respectively. **i** the competition factor $\theta = \frac{\tau_{ext}}{\tau}$ derived as a function of V_{cor} with the short-circuit condition and maximum power output point highlighted in black and red, respectively.

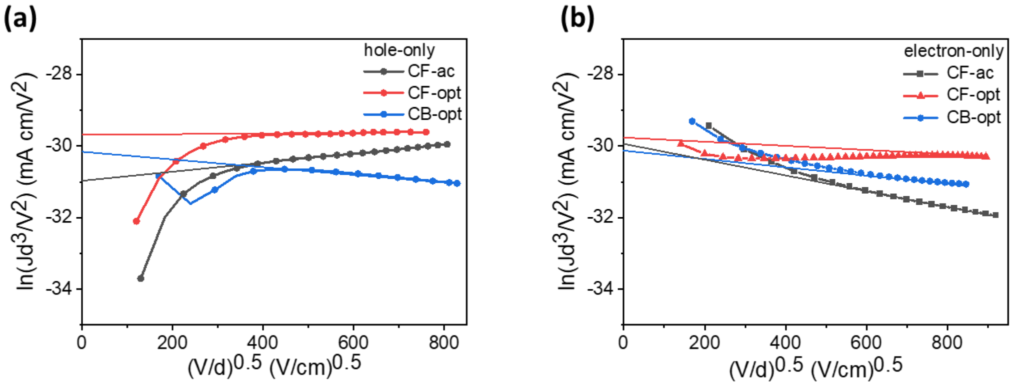

Supplementary Fig. 22 *J*-*V* characteristics in the dark for **a** hole-only and **b** electron-only devices based on PM6:Y6 with different processing conditions.

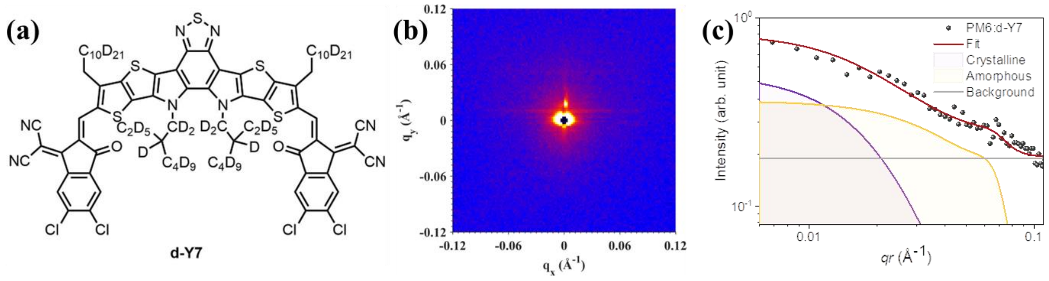

Supplementary Fig. 23 a Molecular structure of *d*-Y7. **b** the 2D GISANS pattern of PM6:*d*-Y7 blend films and **c** the corresponding horizontal linecut along the in-plane direction (dots) with its best fit (solid line).

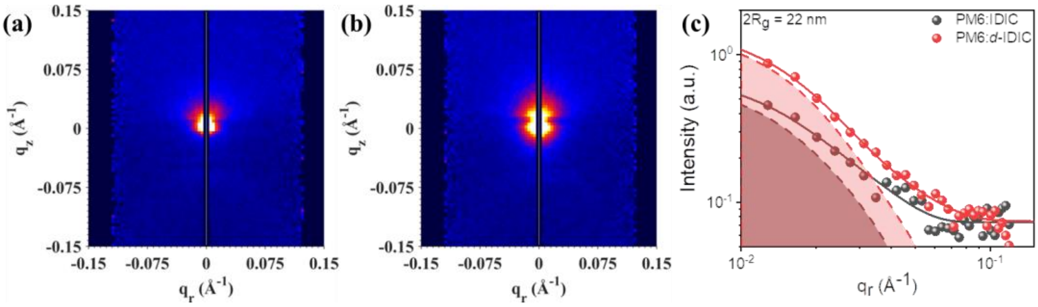

Supplementary Fig. 24 2D GISANS patterns of **a** PM6:IDIC, **b** PM6:*d*-IDIC blend films and **c** corresponding horizontal linecuts along the in-plane direction (dots) with their best fittings (solid line).

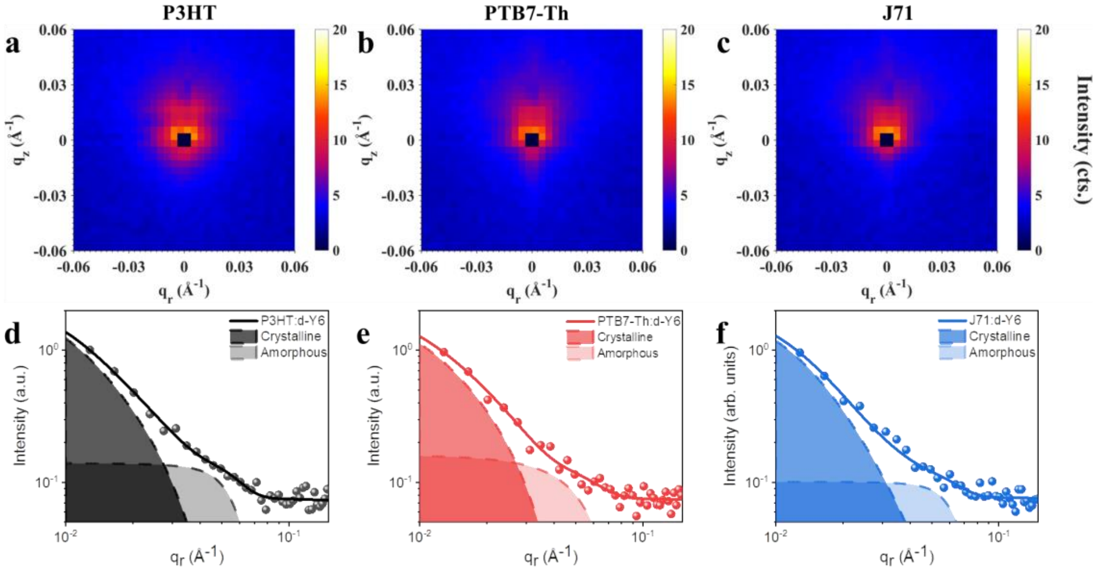

Supplementary Fig. 25 2D GISANS patterns of **a** P3HT: *d*-Y6, **b** PTB7-Th: *d*-Y6, and **c** J71: *d*-Y6, with their corresponding horizontal linecuts (dots) and best fittings (solid line) shown in **d**-**f**.

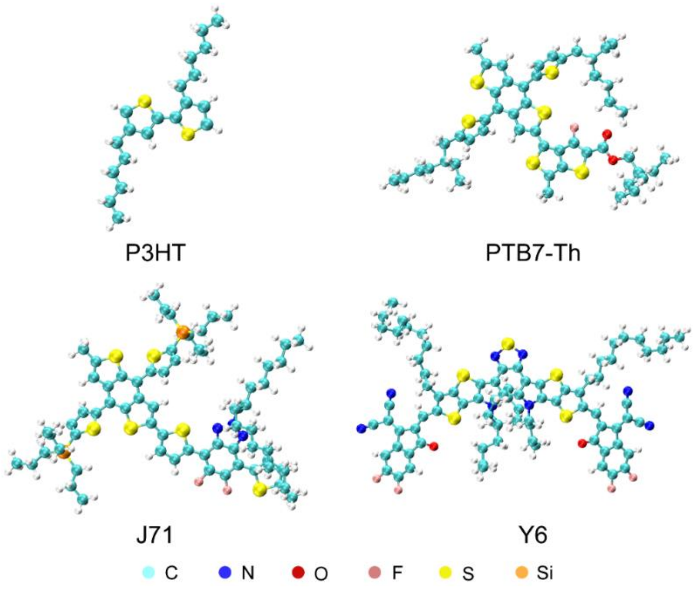

Supplementary Fig. 26 Molecular structures of the P3HT, PTB7-Th, J71 monomers and Y6 molecule.

Supplementary Tables

Supplementary Table 1 SLD values of pure and blend films under the X-ray.

Sample	Critical angle (°)	SLD (Å^{-2})
PM6	0.211	1.76×10^{-5}
Y6	0.207	1.74×10^{-5}
d-Y6	0.207	1.69×10^{-5}
PM6:Y6	0.204	1.72×10^{-5}
PM6:*d*-Y6	0.205	1.75×10^{-5}

Supplementary Table 2 SLD values of pure and blend films under the neutron beam.

Sample	SLD (Å^{-2})

PM6	(1.2±0.1)×10^{-6}
Y6	(2.3±0.1)×10^{-6}
d-Y6	(6.4±0.4)×10^{-6}
PM6:Y6	(1.7±0.1)×10^{-6}
PM6:*d*-Y6	(3.0±0.2)×10^{-6}

Supplementary Table 3 A summary of device characteristics of PM6:Y6 and PM6:*d*-Y6 systems under three different processing conditions mentioned in the main text.

Blends	*Voc (V)* [d]	*Jsc (mA·cm^{-2})* [d]	*FF (%)* [d]	*PCE (%)* [d]
PM6: Y6 [a]	0.865 (0.861±0.003)	26.9 (26.7±0.2)	69.7 (69.0±0.5)	16.3 (15.9±0.3)
PM6: d-Y6 [a]	0.856 (0.855±0.003)	27.1 (26.7±0.3)	67.2 (66.8±0.5)	15.6 (15.2±0.2)
PM6: Y6 [b]	0.853 (0.850±0.002)	28.0 (27.7±0.5)	74.4 (74.1±0.4)	17.8 (17.4±0.3)
PM6: d-Y6 [b]	0.846 (0.843±0.002)	27.7 (27.7±0.2)	75.1 (74.7±0.3)	17.6 (17.4±0.2)
PM6: Y6 [c]	0.806 (0.798±0.006)	25.5 (24.7±0.6)	72.2 (71.5±0.5)	14.8 (14.1±0.5)
PM6: d-Y6 [c]	0.807 (0.807±0.003)	24.9 (24.6±0.5)	72.1 (72.0±0.4)	14.5 (14.3±0.2)

[a] As-cast w/o CN & TA. [b] CF-opt (w/ 0.5% CN & TA 90℃ 5min). [c] CB-opt (w/ 0.5% CN & TA 90℃ 5min). [d] Average values with their standard deviations (in parentheses) are obtained from 5 independent devices.

Supplementary Table 4 Charge carrier mobilities of PM6:Y6 blend films processed under different conditions measured by the SCLC Method.

Active layer	μ_h (× 10^{-4} cm^2 V^{-1} s^{-1}) [a]	μ_e (× 10^{-4} cm^2 V^{-1} s^{-1}) [a]
CF-ac	2.1±0.3	5.9±0.4
CF-opt	4.7±0.2	4.8±0.3

CB-opt	7.5±0.4	6.8±0.3

[a] Average values are obtained from 5 independent devices.

Supplementary Table 5 Morphology parameters fitted from the GISANS intensity profiles ($2R_{gc}$ is the crystallized acceptor domain size, $2R_{ga}$ is the amorphous acceptor domain size in the intermixing phase.)

Active layer	$2R_{gc}$ (nm)	$2R_{ga}$ (nm)
PM6:*d*-Y7	13.8	5.4
PM6:*d*-IDIC	22	N/A
P3HT:*d*-Y6	33.7	7.5
PTB7-Th:*d*-Y6	31.7	6.7
J71:*d*-Y6	28.7	6.7
