# Supplementary material for: Deuteration-enhanced neutron contrasts to probe amorphous domain sizes in organic photovoltaic bulk heterojunction films
Source: Nat Commun. 2024 Mar 30;15:2784. doi: 10.1038/s41467-024-47052-7 (PMC10981694; doi:10.1038/s41467-024-47052-7)
Supplement: Supplementary file 3 — Description of Additional Supplementary Files [file 41467_2024_47052_MOESM3_ESM.pdf]

Description of Additional Supplementary Files

File name: Supplementary Data 1

Description: Initial atomic configurations for MD simulation of the J71:Y6 blend.

File name: Supplementary Data 2

Description: Optimized atomic configurations for MD simulation of the J71:Y6 blend.

File name: Supplementary Data 3

Description: Initial atomic configurations for MD simulation of the P3HT:Y6 blend.

File name: Supplementary Data 4

Description: Optimized atomic configurations for MD simulation of the P3HT:Y6 blend.

File name: Supplementary Data 5

Description: Initial atomic configurations for MD simulation of the PTB7:Y6 blend.

File name: Supplementary Data 6

Description: Optimized atomic configurations for MD simulation of the PTB7:Y6 blend.
