# Supplementary material for: Deuteration-enhanced neutron contrasts to probe amorphous domain sizes in organic photovoltaic bulk heterojunction films
Source: Nat Commun. 2024 Mar 30;15:2784. doi: 10.1038/s41467-024-47052-7 (PMC10981694; doi:10.1038/s41467-024-47052-7)
Supplement: Supplementary file 10 — Reporting Summary [file 41467_2024_47052_MOESM10_ESM.pdf]

Corresponding author(s): Xinhui Lu

Solar Cells Reporting Summary

Nature Portfolio wishes to improve the reproducibility of the work that we publish. This form is intended for publication with all accepted papers reporting the characterization of photovoltaic devices and provides structure for consistency and transparency in reporting. Some list items might not apply to an individual manuscript, but all fields must be completed for clarity.

For further information on Nature Research policies, including our data availability policy, see Authors & Referees.

Experimental design

Please check the following details are reported in the manuscript, and provide a brief description or explanation where applicable.

1. Dimensions

Area of the tested solar cells

- [x] Yes — 0.04 cm^2
- [ ] No — *Explain why this information is not reported/not relevant.*

Method used to determine the device area

- [x] Yes — Device area is determined using optical microscope
- [ ] No — *Explain why this information is not reported/not relevant.*

2. Current-voltage characterization

Current density-voltage (J-V) plots in both forward and backward direction

- [ ] Yes
- [x] No — OPV devices do not show notable hysteresis, so only forward scans were measured

Voltage scan conditions

- [x] Yes — From -0.2 V to 1.2 V with a 20 mV step and a dwell time of 10 ms
- [ ] No — *Explain why this information is not reported/not relevant.*

Test environment

- [x] Yes — All devices are tested in a nitrogen-filled glove box at low (~0.02 ppm) humidity at room temperature
- [ ] No — *Explain why this information is not reported/not relevant.*

Protocol for preconditioning of the device before its characterization

- [ ] Yes — *Provide a description of the protocol.*
- [x] No — It has negligible effect on our J-V curves measured

Stability of the J-V characteristic

- [ ] Yes — *Provide a description of the method used. The stability of the J-V characteristic can be verified with time evolution of the maximum power point or with the photocurrent at maximum power point; see ref. 5 for details.*
- [x] No — Stability is not the main focus of this manuscript.

3. Hysteresis or any other unusual behaviour

Description of the unusual behaviour observed during the characterization

- [ ] Yes — *Provide a description of hysteresis or any other unusual behaviour observed during the characterization.*
- [x] No — Not applicable for the scope of this manuscript

Related experimental data

- [ ] Yes — *Provide a description of the related experimental data.*
- [x] No — Not applicable

4. Efficiency

External quantum efficiency (EQE) or incident photons to current efficiency (IPCE)

- [ ] Yes — *Provide a description of the technique used.*
- [x] No — This manuscript focused on morphology characterization of reported systems instead of exploring new systems or reporting record efficiencies

A comparison between the integrated response under the standard reference spectrum and the response measure under the simulator

- [ ] Yes — *State where this information can be found in the text.*
- [x] No — Not applicable

For tandem solar cells, the bias illumination and bias voltage used for each subcell

☐ Yes — *Provide a description of the measurement conditions.*
☒ No — Not applicable

5. Calibration

Light source and reference cell or sensor used for the characterization

☒ Yes — Method section in the manuscript
☐ No — *Explain why this information is not reported/not relevant.*

Confirmation that the reference cell was calibrated and certified

☒ Yes — Method section in the manuscript
☐ No — *Explain why this information is not reported/not relevant.*

Calculation of spectral mismatch between the reference cell and the devices under test

☐ Yes — *Provide a value of the spectral mismatch and/or a description of how it has been taken into account in the measurements.*
☒ No — Not applicable

6. Mask/aperture

Size of the mask/aperture used during testing

☐ Yes — *Report the size of the mask/aperture.*
☒ No — No mask was used

Variation of the measured short-circuit current density with the mask/aperture area

☐ Yes — *Report the difference in the short-circuit current density values measured with the mask and aperture area.*
☒ No — Not applicable

7. Performance certification

Identity of the independent certification laboratory that confirmed the photovoltaic performance

☐ Yes — *Identify the independent certification laboratory.*
☒ No — Not applicable

A copy of any certificate(s)

☐ Yes — *Certificate copies should be provided in the Supplementary information. Please state the supplementary item number.*
☒ No — Not applicable

8. Statistics

Number of solar cells tested

☒ Yes — 15 independent cells on around 5 independent substrates were measured for each condition
☐ No — *Explain why this information is not reported/not relevant.*

Statistical analysis of the device performance

☒ Yes — For each parameter, the average and the highest values, as well as the standard deviations were provided
☐ No — *Explain why this information is not reported/not relevant.*

9. Long-term stability analysis

Type of analysis, bias conditions and environmental conditions

☐ Yes — *Provide a description of the type of analysis, bias conditions and environmental conditions (e.g. illumination type, temperature, atmosphere humidity, encapsulation method, preconditioning temperature, bias) for each long-term stability analysis carried out; see ref. 7 and 8 for details.*
☒ No — Not applicable for the scope of this manuscript
